# Vibrational Spectroscopy of Tungsten(VI) Chlorides: WCl_6_ and WOCl_4_

**DOI:** 10.1002/open.202500338

**Published:** 2025-09-26

**Authors:** Stewart F. Parker, Talha Nasir

**Affiliations:** ^1^ ISIS Neutron and Muon Facility STFC Rutherford Appleton Laboratory Chilton OX11 0QX UK

**Keywords:** density functional theory, inelastic neutron scattering, infrared spectroscopy, Raman spectroscopy, tungsten chloride

## Abstract

Tungsten (VI) hexachloride, WCl_6_, and tungsten (VI) oxytetrachloride, WOCl_4_, are of interest in a variety of technological applications. In the present work, complete vibrational assignments are provided for both molecules in the “free” state (i.e., in the gas phase or in solution) and in the solid state. The first inelastic neutron scattering spectra of both molecules in the solid state are recorded. The assignments are supported by density functional theory calculations. In all cases, the W–Cl stretching modes occur in the 300–400 cm^−1^ range, and the Cl–W–Cl bending modes in the 100‐250 cm^−1^ range.

## Graphical abstract

WCL_6_ (left) and WOCl_4_ (right) are used for a variety of processes. Complete vibrational assignments are provided for both molecules as isolated systems and in the solid state. The assignments are based on new infrared, Raman, and the first inelastic neutron scattering spectra, supported by density functional theory calculations.

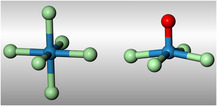

## 1 Introduction

Tungsten and chlorine form a wide range of binary (WCl_6_, WCl_5_, WCl_4_, “WCl_3_”, and “WCl_2_”) and ternary (WOCl_4_, WO_2_Cl_2_, WOCl_3_, and WOCl_2_) compounds.^[^ ^1^ ^]^ WCl_6_ is used as a catalyst in a variety of reactions, including alkene^[^ ^2^ ^]^ and alkyne^[^ ^3^ ^]^ polymerization, alkene metathesis,^[^ ^4^ ^]^ dithioacetalization of carbonyl compounds,^[^ ^5^ ^]^ and ring‐opening polymerization.^[^ ^6^ ^]^ WCl_6_ is also used as a source of tungsten for a variety of surface coatings, including W metal,^[^ ^7^ ^]^ WC,^[^ ^8^ ^]^ and WO_3_.^[^ ^9^ ^]^ WOCl_4_ has been proposed as a candidate material for the cathode in rechargeable chloride‐ion batteries.^[^ ^10^ ^]^

The structures of the compounds have been extensively investigated. In the gas,^[^ ^11^ ^]^ liquid,^[^ ^12^ ^]^ and solid phase^[^ ^13^ ^,^ ^14^ ^]^ WCl_6_ is molecular and octahedral. WCl_5_ is trigonal bipyramidal in the gas phase^[^ ^15^ ^]^ but dimerizes in the solid state to give W_2_Cl_10_ ([WCl_5_(μ‐Cl)]_2_), with distorted edge‐sharing octahedra.^[^ ^16^ ^]^ On volatilization of WCl_4_, mass spectroscopy shows the presence of significant amounts of WCl_5_, WCl_4_, the dimeric trichloride, W_2_Cl_6_, and small amounts of the trimer W_3_Cl_9_.^[^ ^17^ ^]^ Gas electron diffraction^[^ ^18^ ^]^ suggests a distorted tetrahedral structure, *D*_2d_, but computational studies indicate a regular tetrahedron, *T*_d_.^[^ ^19^ ^]^ In the solid state, it forms infinite linear polymers. The coordination is octahedral: each W atom is surrounded by four bridging and two terminal Cl atoms in trans positions, [WCl_2_(µ‐Cl)_2_]_∞_.^[^ ^20^ ^]^ For WCl_3_, as with WCl_4_, there is a conflict between experiment and theory for the gas phase structure: gas electron diffraction^[^ ^18^ ^]^ suggests a T‐shaped structure, but theory finds a planar, symmetric *D*_3h_ structure as the ground state.^[^ ^19^ ^]^ The solid state structure is unexpected: WCl_3_ forms hexamers in the solid state, six tungsten atoms being situated at the corners of a perfect octahedron, each metal atom carries one terminal Cl atom; the remaining twelve bridge the octahedral edges, [W_6_Cl_6_(µ‐Cl)_12_].^[^ ^21^ ^]^ Thus, each W is five‐coordinated but with a square‐based pyramidal coordination (i.e., *D*_4h_). The solid state structure of WCl_2_ has not been determined, but it is said to be isotypic with MoCl_2_.^[^ ^22^ ^]^ WCl_2_ is also a hexamer; the W atoms occupy positions at the corners of an octahedron; each of the eight octahedral faces is capped by a triply bridging Cl atom. Two W atoms in trans positions carry terminal Cl atoms, and the four equatorial W atoms are linked to neighboring octahedral units through Cl bridges, {[W_6_Cl_2_(µ^3^‐Cl)_8_](µ‐Cl)_2_}_∞_.

Of the oxychlorides, WOCl_4_ is molecular in the gas phase.^[^ ^23^ ^]^ In the solid state, each tungsten atom is surrounded by two oxygen atoms and four chlorine atoms in a distorted octahedron. The octahedra are linked via oxygen bridges to chains that run parallel to the *c*‐axis.^[^ ^24^ ^]^ WO_2_Cl_2_ extends this motif into two dimensions.^[^ ^25^ ^]^ The octahedra are again distorted, with the Cl in the axial positions and the bridging O in the equatorial plane forming a 2D layer. WOCl_3_ consists of pairs of edge‐sharing WO_2_Cl_4_ octahedra, which are connected via oxygen bridges to form infinite chains along the *c*‐axis.^[^ ^26^ ^]^ WOCl_2_ extends this motif: the O atoms bridge W atoms and the Cl atoms are edge sharing, thus there is octahedral coordination of the W, i.e., “WO_2_Cl_4_”.^[^ ^27^ ^]^

While the structural chemistry of the W/Cl/O system has been comprehensively studied, this is not the case for the vibrational spectroscopy. WCl_6_ is the best studied; infrared and Raman data for the gaseous and solid states are available.^[^ ^23^ ^,^ ^28^, ^29^, ^30^ ^–^ ^31^ ^]^ WOCl_4_ has been studied in the gas, matrix, and solid states,^[^ ^23^ ^,^ ^32^, ^33^ ^–^ ^34^ ^]^ and except for one report^[^ ^23^ ^]^ of the gas phase Raman spectrum, we are unaware of any data on WCl_4_. To the best of our knowledge, the other compounds have not been studied spectroscopically. This is probably because only WCl_6_, WCl_4_, and WOCl_4_ are commercially available.

Even for the most studied compound, WCl_6_, the data are incomplete because the lowest energy bending mode is forbidden in both the infrared and the Raman spectrum under *O*_h_ symmetry. Even if the site symmetry is lower than *O*_h_ and the mode becomes formally allowed, it generally has very low intensity, making its detection very difficult. Inelastic neutron scattering (INS) spectroscopy is a complementary form of vibrational spectroscopy where there are no selection rules.^[^ ^35^ ^]^ We have previously shown that it is readily able to observe all of the modes, both internal and external, in hexahalo‐metal complexes,^[^ ^36^ ^]^ and the same methodology will apply to WCl_6_. In the present work, we present the first INS spectra of WCl_6_ and WOCl_4_ together with new infrared and Raman spectra of the compounds in solution and in the solid state. Our assignments are supported by density functional theory (DFT) calculations.

## 2 Results and Discussion

The compounds WCl_6_ and WOCl_4_ exhibit a variety of structural motifs. In the gas phase (and presumably also in solution), they are monomeric, while in the solid state, they exhibit octahedral coordination, albeit through different means. In the following, we will discuss each of the compounds as both the “free” molecule and in the solid state.

### 2.1 Tungsten Hexachloride, WCl_6_

**Figure 1:**
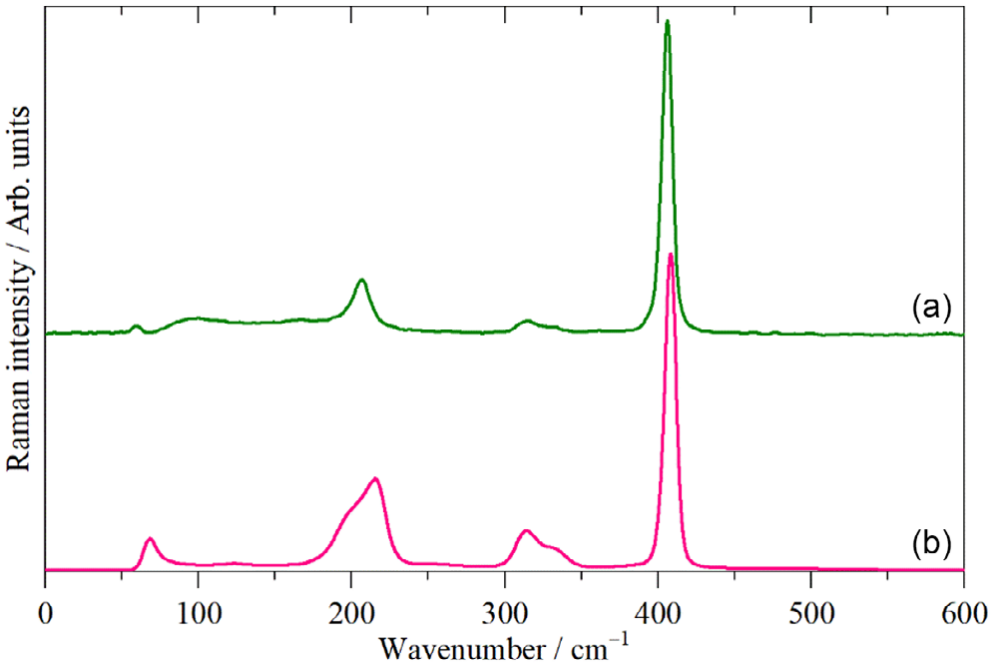
FT‐Raman spectra of WCl_6_ in a) CS_2_ solution (solvent subtracted) and b) the solid state.

WCl_6_ is monomeric in the gas, solution, liquid, and solid state. **Figure** **1** shows the Raman spectrum of WCl_6_ in CS_2_ solution and in the solid state. Apart from some intensity changes, it is apparent that both spectra are the same, as expected for an unchanged structure.

Under *O*_h_ symmetry, the vibrations of WCl_6_ are classified as W–Cl stretch: *A*_1g_ (*ν*_1_), *E*_g_ (*ν*_2_), *T*_1u_ (*ν*_3_), and the Cl–W–Cl bends as *T*_1u_ (*ν*_4_), *T*_2g_ (*ν*_5_), and *T*_2u_ (*ν*_6_). The gerade (g) modes are Raman allowed, *T*_1u_ is infrared allowed, and *T*_2u_ is forbidden in both the Raman and the infrared. The selection rules mean that the assignment is unambiguous except for the unseen mode *ν*_6_. From a combination mode, this has been proposed to be at 97 cm^−1^.^[^ ^28^ ^]^ Our CASTEP calculation and one using GAUSSIAN^[^ ^19^ ^]^ predict *ν*_6_ at 86 and 87 cm^−1^, respectively, in reasonable agreement. **Table** **1** lists the observed and calculated transition energies.

**Table 1:** Experimental and calculated transition energies for molecular WCl_6_.

DFT/cm^−1^	Infrared intensity/km mol^−1^	Raman intensity/Å^4^ amu^−1^	Ramana)/cm^−1^ CS_2_ sol’nb)	Gasphase^[^ ^23^ ^]^	Infrared/cm^−1^ CCl_4_ sol’n^[^ ^28^ ^]^	Gasphase^[^ ^31^ ^]^	Description
86 (*T*_2u_)							Cl–W–Cl bend (*ν*_6_)
163 (*T*_1u_)	17.19				165s	161s	Cl–W–Cl bend (*ν*_4_)
192 (*T*_2g_)		6.67	207m	205w			Cl–W–Cl bend (*ν*_5_)
313 (*E*_g_)		24.00	314w	313w			W–Cl stretch (*ν*_2_)
353 (*T*_1u_)	142.40				367vs	366vs	W–Cl stretch (*ν*_3_)
387 (*A*_g_)		69.95	406s	404vs			W–Cl stretch (*ν*_1_)

^a)^ s = sharp, m = medium, w = weak, sh = shoulder, br = broad, v = very.

^b)^ This work

In the solid state. WCl_6_ exists as two polymorphs: *α* and *β*. *α*‐WCl_6_ is rhombohedral, space group $R\bar{3}$ ($C_{3i}^{2}$, no. 148) with *Z* = 1 in the primitive cell.^[^ ^37^ ^]^ This is the usual form at room temperature, and powder X‐ray diffraction confirms that our sample is *α*‐phase (see Figure S1, Supporting Information). Heating *α*‐WCl_6_ to 176 °C transforms it to *β*‐WCl_6_. This is a hexagonal, space group $P\bar{3}m1$ ($D_{3d}^{3}$no. 164) with Z = 3 in the primitive cell. It is not clear whether the phase change is irreversible or whether *β*‐WCl_6_ is metastable, but it can be cooled to at least 133 K and retain the structure.^[^ ^13^ ^]^ Our DFT calculations show *α*‐WCl_6_ as the lowest energy structure (at 0 K), but the difference between the two polymorphs is only 0.011 eV, so to within the accuracy of DFT, the two structures are isoenergetic.

**Figure 2:**
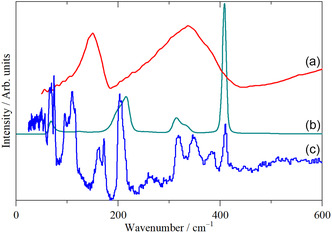
Vibrational spectra of WCl_6_ in the solid state: a) infrared spectrum, b) FT‐Raman spectrum, and c) INS spectrum. (a), (b) are recorded at room temperature, and (c) at 10 K.

**Figure** **2** shows the infrared, Raman, and INS spectra of WCl_6_ in the solid state. It is apparent that all three forms of spectroscopy are essential for a complete assignment.

For *α*‐WCl_6_, the site symmetry is *C*_3i_, and the correlation table^[^ ^38^ ^]^ is shown in Table S2, Supporting Information. It can be seen that the effect of the symmetry reduction is that all of the *T* bands split to an (*A *+ *E*) doublet; however, because the center of symmetry is retained, the selection rules remain largely the same, although *ν*_6_ is now formally allowed in the infrared spectrum but would be expected to be very weak. Note that all modes are INS allowed. Hence, the assignment is straightforward. The intense infrared bands at 337 and 149 cm^−1^ are *ν*_3_ and *ν*_4_, respectively. The Raman bands at 406, 314, and 206 cm^−1^ are *ν*_1_ (*A*_1g_,), *ν*_2_ (*E*_g_), and *ν*_5_ (*T*_2g_), respectively. The INS spectrum shows *ν*_1_ and *ν*_2_ at the same energies as the Raman spectrum. The much narrower band widths of the INS spectrum as compared to the infrared allow both components of the split *ν*_3_ (*T*_1u_) to be observed at 384 and 348 cm^−1^ with ≈2:1 intensity ratio, confirming them as the *E*_*u*_ and *A*_*u*_ components, respectively. Large bandwidths for the infrared modes are a common feature of the spectra of hexahalo metal ions. This presumably is a result of strong electrical anharmonicity, an effect that has no consequences for the INS spectrum, as the intensity derives from the amplitude of motion and the neutron scattering cross section. *ν*_5_ (*T*_2g_) and *ν*_4_ (*T*_1u_) are seen at approximately the same energies as in the Raman and infrared spectra respectively; the small difference (see **Table** **2**) is probably a result of the infrared and Raman spectra being measured at room temperature and the INS at ≈10 K. The feature at 100 cm^−1^ that is present in neither the infrared nor Raman spectrum is the optically forbidden mode *ν*_6_ (*T*_2u_). The Raman allowed (*T*_1g_) librational mode is observed at 67 cm^−1^, and the acoustic translational modes comprise the broad feature peaking at ≈50 cm^−1^. INS spectroscopy is sensitive to modes at any wavevector (a result of the neutron having mass), whereas infrared and Raman spectra are only seen at ≈0 wavevector (the Brillouin zone *Γ*‐point). The acoustic modes have 0 energy at the *Γ*‐point, so are not observed by infrared or Raman spectroscopy.

**Table 2:** Experimental and calculated (at the *Γ*‐point) transition energies for *α*‐WCl_6_.

DFT/cm^−1^	Infrared intensity/km mol^−1^	Raman intensity/Å^4^ amu^−1^	INSa)/cm^−1^	Raman/cm^−1^	Infrared/cm^−1^	Description
0 (*E*_u_)			39m, br	0	0	Acoustic mode
0 (*A*_u_)				0	0	Acoustic mode
66 (*E*_g_)		30.95	68s	68m		Libration
81 (*A*_g_)		13.00	75m			Libration
102 (*E*_u_)	0.61		103s, br			Cl–W–Cl bend (*ν*_6_)
122 (*A*_u_)	0.01		110m			Cl–W–Cl bend (*ν*_6_)
158 (*E*_u_)	102.96		163m		150s, br	Cl–W–Cl bend (*ν*_4_)
163 (*A*_u_)	72.48		172m			Cl–W–Cl bend (*ν*_4_)
201 (*E*_g_)		342.48	204s	202sh		Cl–W–Cl bend (*ν*_5_)
208 (*A*_g_)		592.95	216sh	216s		Cl–W–Cl bend (*ν*_5_)
			267w, br			Combination mode (*ν*_6_ + *ν*_4_)
319 (*E*_g_)		437.72	318m	314m		W–Cl stretch (*ν*_2_)
				333sh		Combination mode (*ν*_6_ + *ν*_5_)
331 (*E*_u_)	493.21		349s, br		338vs, vbr	W–Cl stretch (*ν*_3_)
338 (*A*_u_)	442.52		384m			W–Cl stretch (*ν*_3_)
398 (*A*_g_)		2682.68	410m	409vs		W–Cl stretch (*ν*_1_)

^a)^ s = sharp, m = medium, w = weak, sh = shoulder, br = broad, v = very.

Based on experimental spectra, we have previously derived a correlation between *ν*_5_ and *ν*_6_ that is accurate to better than 10%: *ν*_6_ = 18 + 0.63 *ν*_5_.^[^ ^36^ ^]^ This predicts *ν*_6_ at 148 cm^−1^ rather than 112 cm^−1^ (average of factor group components). WCl_6_ is unusual in that *ν*_5_ > *ν*_4_; almost all other systems are the reverse.^[^ ^36^ ^]^ If *ν*_4_ is used rather than *ν*_5_ in the correlation, the predicted transition energy is 120 cm^−1^, within the 10% error bar. This suggests a modification to the correlation; it is the lower of *ν*_4_ and *ν*_5_ that should be used, rather than always *ν*_5._

The shoulder present on the high energy side of *ν*_2_ coincides with *ν*_3_, but this is incompatible with *O*_h_ symmetry and the INS spectrum. We assign this as a combination mode (*ν*_6_ + *ν*_5_) in Fermi resonance with *ν*_2_.

**Figure 3:**
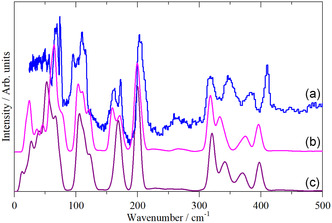
Comparison of: a) the INS spectrum of WCl_6_ with those calculated for, b) *α*‐WCl_6_, and c) *β*‐WCl_6_.

DFT calculations confirm these assignments, see Table 2. **Figure** **3** shows a comparison of the observed INS spectrum and that calculated for both the *α* and *β* phases. It is apparent that the *α* phase is the better match, as expected. The spectrum of the *β* phase is more complex because there are three molecules in the primitive cell on two different sites (*D*_3d_ and *C*_3v;_ the correlation table is shown in Table S2, Supporting Information). Both calculations are for the complete Brillouin zone; the dispersion (variation of transition energy with wavevector) curves are shown in Figure S2, S3, Supporting Information. As would be expected for systems where there is little interaction between molecules, the dispersion curves are nearly flat, consistent with the sharp bands in the INS spectrum.

### 2.2 Tungsten Oxytetrachloride, WOCl_4_

In the gas phase, WOCl_4_ is monomeric with a square pyramidal *C*_4v_ structure as shown by gas electron diffraction.^[^ ^39^ ^]^ The W atom lies above the plane of the four Cl atoms with ∠OWCl = 102.4 ± 1.4°. An isolated *C*_4v_ WOCl_4_ molecule has nine modes: there is one W = O stretch (*A*_1_), three W–Cl stretch modes (*A*_1_ *, B*_1_, and *E*), two in‐plane Cl–W–Cl bending modes (*B*_2_ and *E*), and three out‐of‐plane bending modes (*A*_1_ *, B*_1_, and *E*). The form of the normal modes is shown elsewhere.^[^ ^40^ ^]^

**Figure 4:**
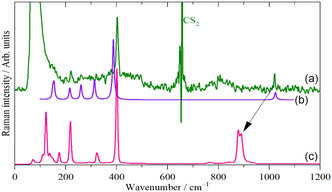
FT‐Raman spectra of WOCl_4_ in a) CS_2_ solution (solvent subtracted), b) calculated raman spectrum for the isolated molecule, and c) the solid state.

**Figure** **4a** shows the Raman spectrum of WOCl_4_ in CS_2_ solution (solvent subtracted), Figure 4b shows the calculated Raman spectrum for the isolated molecule at 300 K. **Table** **3** lists the calculated and observed^[^ ^23^ ^,^ ^32^ ^]^ transition energies; it is clear that the agreement between theory and experiment for both transition energy and intensity is excellent.

**Table 3:** Experimental and calculated transition energies for molecular WOCl_4_ (gas phase and solution).

DFT/cm^−1^	Infrared intensity/km mol^−1^	Raman intensity/Å^4^ amu^−1^	Ramana)/cm^−1^CS_2_ sol’nb)	Gas phase^[^ ^32^ ^]^	Gas phase^[^ ^23^ ^]^	Infrared/cm^−1^Gas phase^[^ ^32^ ^]^	Description
41 (*B*_1_)		1.95					oop Cl–W–Cl bend (*ν*_5_)
150 (*E*)	13.42	0.70					ip Cl–W–Cl bend (*ν*_9_)
154 (*A*_1_)	0.27	2.41	152w		153w		oop Cl–W–Cl bend (*ν*_3_)
217 (*B*_2_)		3.53	221w				ip Cl–W–Cl bend (*ν*_6_)
261 (*E*)	1.07	3.02			256w		Cl–W=O bend (*ν*_8_)
313 (*B*_1_)		10.94					W–Cl stretch (*ν*_4_)
372 (*E*)	131.59	0.14				383s	W–Cl stretch (*ν*_7_)
388 (*A*_1_)	4.24	46.06	402s	402vs	404s	404vw	W–Cl stretch (*ν*_2_)
1024 (*A*_1_)	72.99	24.33	1021w	1027w	1027w	1027s	W=O stretch (*ν*_1_)

^a)^ s = sharp, m = medium, w = weak, sh = shoulder, br = broad, v = very, oop = out‐of‐plane, ip = in‐plane.

b) This work.

**Figure 5:**
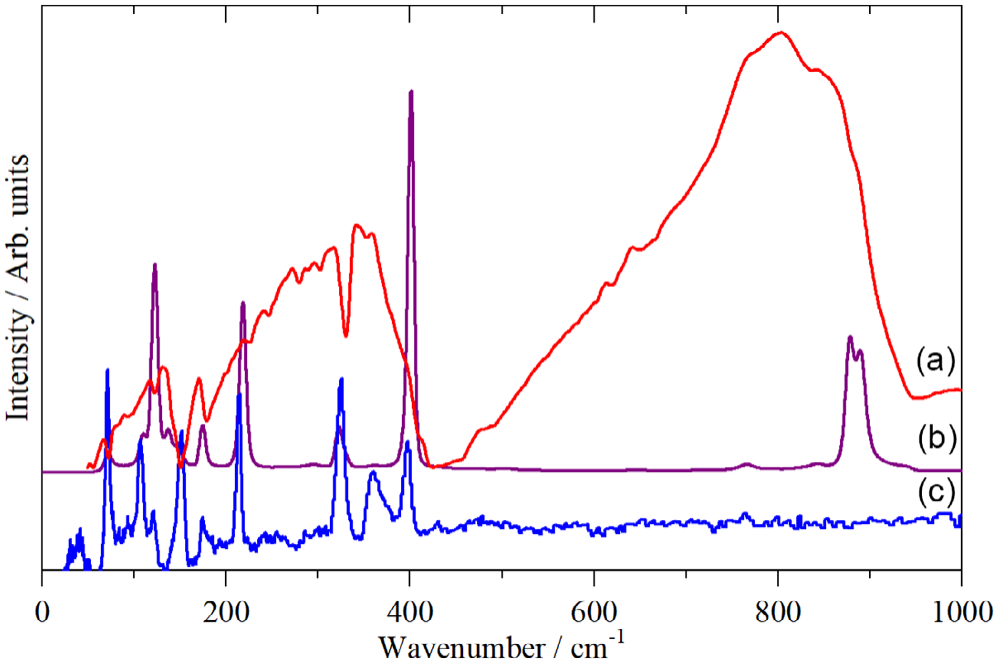
Vibrational spectra of WOCl_4_ in the solid state: a) infrared spectrum, b) FT‐Raman spectrum, and c) INS spectrum. (a) and (b) are recorded at room temperature and (c) at 10 K.

In the solid state of WOCl_4_ (space group *I*4, ($C_{4}^{5}$, no. 79)) the molecules are linked via asymmetric O—W—O bonds with O—W distances of 1.739 and 2.231 Å.^[^ ^24^ ^]^ This results in distorted octahedral coordination around the W atom; the W is above the plane of the four Cl atoms as in the gas phase, albeit with ∠OWCl = 98.5°. Under the *C*_4_ factor group, the symmetry labels are the same as for the isolated *C*_4v_ molecule, except that the subscripts are dropped. The correlation table is shown in Table S3, Supporting Information. All modes are INS and Raman allowed, *A* and *E* modes are infrared allowed. The solid state spectra of WOCl_4_ have been investigated by Raman spectroscopy,^[^ ^32^ ^,^ ^34^ ^]^ and we are unaware of any infrared or INS spectra. **Figure** **5** shows the infrared, Raman, and INS spectra of WOCl_4_ in the solid state. Our Raman data are in good agreement with previous work.^[^ ^32^ ^,^ ^34^ ^]^

**Figure 6:**
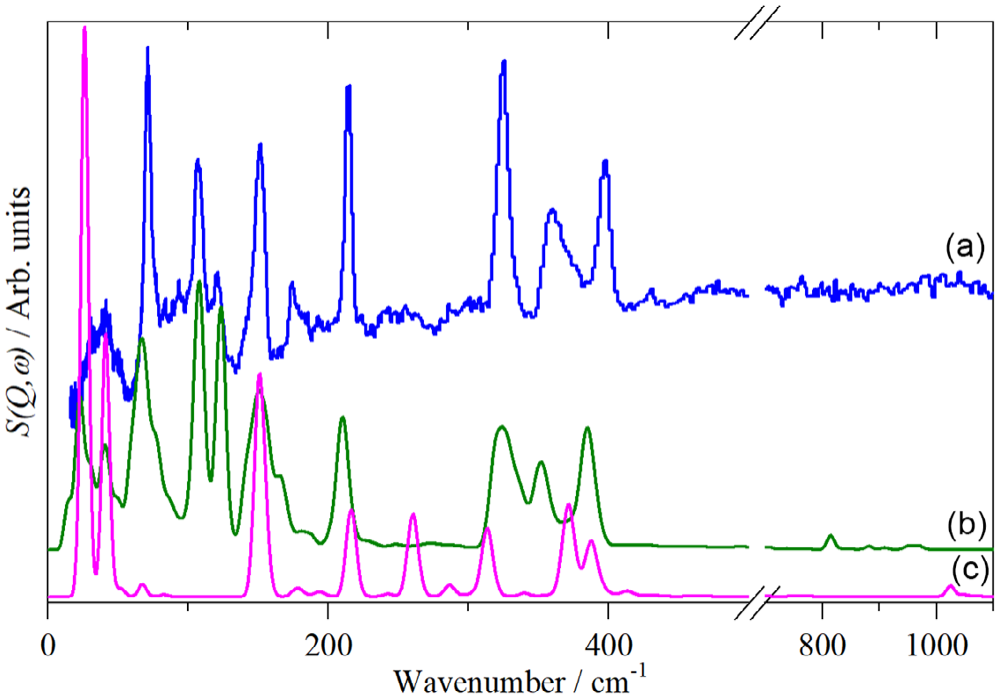
INS spectra of: a) WOCl_4_ in the solid state, b) generated from a fully periodic calculation of the solid, and c) generated from an isolated molecule calculation.

The W = O stretch is clearly seen in the infrared and Raman spectra as the intense band at 800—900 cm^−1^. It is not seen in the INS because it largely involves motion of the W and O atoms, neither of which has any significant cross section (the modes involving Cl motion are easily seen because ^35^Cl has a cross section of 21.86 barn, c.f. O 4.23, W 4.61 barn, 1 barn = 10^−28 ^m^2^). The intense Raman mode at 402 cm^−1^ is the totally symmetric stretch. For the remaining modes, we rely on the DFT calculations. **Figure** **6** shows a comparison of the experimental INS spectrum and those calculated for the solid state structure and the isolated (i.e., gas phase) molecule. It is apparent that there is excellent agreement between the experimental and calculated solid‐state structure, and also that the gas phase to solid state change has had a marked effect on the spectra. The DFT calculations enable a complete assignment to be made, **Table** **4**. The most striking feature is the shift in the W–O modes. As seen in Figure 4a and 4c, the W–O stretching vibration shifts down from 1021 cm^−1^ in CS_2_ solution (1027 cm^−1^ gas phase^[^ ^23^ ^,^ ^32^ ^]^) to 889 cm^−1^ in the solid and the W–O bending (*ν*_9_) mode moves from 256 cm^−1[^ ^23^ ^]^ (261 cm^−1^ calc.) in the isolated molecule to 325 cm^−1^ (331 cm^−1^ calc.) in the solid, consistent with the increased bonding. *ν*_9_ is now coincident with the out‐of‐plane bending mode *ν*_4_, which accounts for the intensity of the 325 cm^−1^ feature, and its width is greater than that of *ν*_2_ at 402 cm^−1^ (on TOSCA, the resolution degrades with increasing energy transfer). The W–Cl modes are almost unchanged on going from solution to solid.

**Table 4:** Experimental and calculated (at the *Γ*‐point) transition energies for WOCl_4_ in the solid state.

DFT/cm^−1^	Infrared intensity/km mol^−1^	Raman intensity/Å^4^ amu^−1^	INSa)/cm^−1^	Raman/cm^−1^	Infrared/cm^−1^	Description
0 (*E*)			38m, br	0	0	Acoustic mode
0 (*A*)				0	0	Acoustic mode
81 (*A*)	0.06	5.47	71s	71w		Libration
108 (*B*)	0.00	12.57	107s			oop Cl–W–Cl bend (*ν*_5_)
124 (*E*)	4.01	47.75	121w	122s		Libration
141 (*E*)	27.00	11.22	151s	149w		oop Cl–W–Cl bend (*ν*_8_)
163 (*A*)	29.56	68.48	163w	163m	171s	oop Cl–W–Cl bend (*ν*_3_)
215 (*B*)		259.06	215s	216s		ip Cl–W–Cl bend (*ν*_6_)
319 (*B*)		176.73	325	322m		oop Cl–W–Cl bend (*ν*_4_)
331 (*E*)	178.13	28.86	325s			O–W–O bend (*ν*_9_)
354 (*E*)	213.66	2.33	360m, br		353vs, vbr	W–Cl stretch (*ν*_7_)
387 (*A*)	5.80	1024.20	402m	397vs		W–Cl stretch (*ν*_2_)
814 (*A*)	1490.59	3849.12		879s	800vvs, vvbr	W–O stretch (*ν*_1_)
				890s		See text

a) s = sharp, m = medium, w = weak, sh = shoulder, br = broad, v = very, oop = out‐of‐plane, ip = in‐plane.

The doublet in the Raman spectrum for the W–O stretch at 879 and 890 cm^−1^ has attracted attention as possible LO‐TO splitting^[^ ^32^ ^]^ although the temperature dependence rules this out (the high energy feature decreases in intensity with decreasing temperature, an observation we have reproduced). The calculated dispersion curves, Figure S3, Supporting Information, confirm that there is no LO‐TO splitting. The transition energy is too high to be a first overtone (*ν*_2_ is the next highest mode and 2*ν*_2_ = 798 cm^−1^), so it must be a ternary (or higher) mode in Fermi resonance with the W–O stretch. The doublet only occurs in the solid state, so this implies that a mode (or modes) that are only present in the solid state are involved. One candidate is the librational mode at 122 cm^−1^; a combination mode of (lib + *ν*_7_ + *ν*_2_) = 122 + 354 + 397 = 873 cm^−1^ is in reasonable agreement, although this would not explain the temperature dependence.

The calculated dispersion curves, Figure S3, Supporting Information, do show that the W–O stretch mode is strongly dispersive, and as the INS spectrum can be considered to be the projection across the entire Brillouin zone onto the energy axis, this would result in the mode spanning 150 cm^−1^, which also helps explain why it is not seen in the INS spectrum.

## 3 Conclusion

In this work, we have provided the first complete assignments of the vibrational spectroscopy of WCl_6_ and WOCl_4_ in both the isolated molecule and the solid state. In both cases, the W–Cl stretching modes occur in the 300—400 cm^−1^ range and the Cl–W–Cl bending modes in the 100—250 cm^−1^ range. This work emphasizes the need for all three forms of vibrational spectroscopy (infrared, Raman, and INS) in order to make a complete assignment. In particular, the absence of selection rules for the INS spectrum is especially helpful in the low‐energy region. The complementarity of DFT calculations with vibrational spectroscopy is also apparent.

## 4 Experimental Section

4.1

4.1.1

Tungsten(VI) chloride, WCl_6_, and tungsten(VI) oxychloride, WOCl_4_, were purchased from Merck and used as received. Powder X‐ray diffraction of WCl_6_ confirmed that it was the *α*‐phase (see Figure S1, Supporting Information).

INS spectra were recorded at ≈10 K using the TOSCA^[^ ^41^ ^,^ ^42^ ^]^ spectrometer at the ISIS Neutron and Muon Source (Chilton, UK). TOSCA is an indirect geometry spectrometer, so it uses a “white” incident beam, i.e., the full spectral output of the ISIS water moderator. The energy analysis is explained in more detail elsewhere.^[^ ^35^ ^]^ All of the spectra shown are neutron energy loss, so they are on the Stokes side of the elastic line. The spectra are presented as a function of neutron energy loss (in cm^−1^) and the scattering law, *S(Q,ω)* (arbitrary units). An empty aluminum sample can has been subtracted from the measured INS data. Infrared spectra (50–4000 cm^−1^, 4 cm^−1^ resolution, 256 scans with 8× zerofilling) were recorded at room temperature with a Bruker Vertex 70 Fourier transform infrared spectrometer using a Bruker Platinum single reflection attenuated total internal reflection accessory. The FT‐Raman spectrum was recorded at room temperature from the sample inside a quartz cuvette with a Bruker MultiRam spectrometer using 1064nm excitation (500 mW laser power and 256 scans at 4 cm^−1^ resolution with 8× zerofilling).

Dispersion corrected periodic density functional theory (DFT‐D) calculations were carried out with CASTEP^[^ ^43^ ^]^ (versions 21.1 and 23.1). On‐the‐fly generated norm‐conserving pseudopotentials with a plane‐wave cut‐off of 720 eV (WCl_6_) and 1020eV (WOCl_4_) were used with the PBE functional with the Tkatchenko–Scheffler (TS) dispersion correction scheme within the generalized gradient approximation. Brillouin zone sampling of electronic states was performed on a 7 × 7 × 7 (60 k‐points, WCl_6_) and 7 × 7 × 5 (120 k‐points, WOCl_4_) Monkhorst–Pack grids. The equilibrium structure, an essential prerequisite for lattice dynamics calculations, was obtained by BFGS geometry optimization, after which the residual forces were converged to |0.0012| eV Å^−1^ (WCl_6_) and |0.00003| eV Å^−1^ (WOCl_4_). Phonon frequencies were obtained by diagonalization of the dynamical matrix, computed using density‐functional perturbation theory^[^ ^44^ ^]^ to compute the dielectric response and the Born effective charges, and, from these, the mode oscillator strength tensor and infrared absorptivity were calculated. Raman intensities were calculated by a finite displacement method.^[^ ^45^ ^]^ In addition to the calculation of transition energies at zero wavevector, phonon dispersion was also calculated along high symmetry directions throughout the Brillouin zone. For this purpose, dynamical matrices were computed on a regular grid of wavevectors throughout the Brillouin zone, and Fourier interpolation was used to extend the computed grid to the desired fine set of points along the high‐symmetry paths. The atomic displacements in each mode, which are part of the CASTEP output, enable visualization of the modes in Materials Studio^[^ ^46^ ^]^ to aid assignments and are also all that is required to generate the INS spectrum using the program AbINS.^[^ ^47^ ^]^ It is emphasized that, for the calculated spectra and dispersion curves shown, the transition energies have *not* been scaled.

## Supporting Information

The Supporting Information contains the correlation tables for WCl_6_ and WOCl_4_ (Tables S1 and S2), a comparison of the observed XRD pattern with that calculated for the α and β phases (Figure S1) and the dispersion curves for α‐ and β‐WCl_6_ and WOCl_4_ (Figures S2 – S4).

## Conflicts of Interest

The authors declare no conflict of interest.

## Supporting information

Supplementary Material

## Data Availability

The data that support the findings of this study are available from the corresponding author upon reasonable request.
