# Supplementary material for: Vibrational Spectroscopy of Tungsten(VI) Chlorides: WCl6 and WOCl4
Source: ChemistryOpen. 2025 Sep 26;14(12):e202500338. doi: 10.1002/open.202500338 (PMC12680570; doi:10.1002/open.202500338)
Supplement: Supplementary file 1 — Supplementary Material [file OPEN-14-e202500338-s001.pdf]

Supporting information for:

Vibrational spectroscopy of tungsten(VI) chlorides: WCl_6 and $WOCl_4$

Stewart F. Parker*[a] and Talha Nasir[a]

[a] Prof SF Parker, Mr T Nasir
ISIS Neutron and Muon Facility
STFC Rutherford Appleton Laboratory
Chilton
OX11 0QX
UK

E-mail: stewart.parker@stfc.ac.uk

Table of contents

Table S1. Correlation table for α-WCl_6 (space group $R\bar{3}$ (C_{3i}^2) no. 148) 2

Table S2. Correlation table for β-WCl_6 (space group $P\bar{3}m1$ (D_{3d}^3) no. 164) 3

Figure S1. Comparison of powder XRD patterns for WCl_6 4

Figure S2. Phonon dispersion curves for α-WCl_6 5

Figure S3. Phonon dispersion curves for β-WCl_6 6

Table S3. Correlation table for $WOCl_4$ (space group *I*4, no. 79) 7

Figure S4. Phonon dispersion curves for $WOCl_4$ 8

Table S1. Correlation table for α-WCl_6 (space group $R\bar{3}$ (C_{3i}^2) no. 148).

**Free molecule**		**Crystal (Z = 1)**		**Total**[1]
		**Site**[3] **(C_{3i})**	**Factor group (C_{3i})**	
**Sym.**[2]	**Rep.**	**Rep.**		
O_h	A_{1g} (ν_1)	A_g	$A_g + E_g$	$A_g + E_g$
O_h	E_g (ν_2)	E_g	$A_g + E_g$	$A_g + E_g$
O_h	T_{1u} (ν_3)	$A_u + E_u$	$A_u + E_u$	$A_u + E_u$
O_h	T_{1u} (ν_4)	$A_u + E_u$	$A_u + E_u$	$A_u + E_u$
O_h	T_{2g} (ν_5)	$A_g + E_g$	$A_g + E_g$	$A_g + E_g$
O_h	T_{2u} (ν_6)	$A_u + E_u$	$A_u + E_u$	$A_u + E_u$
O_h	T_{1g} (lib)	$A_g + E_g$	$A_g + E_g$	$A_g + E_g$
O_h	T_{1u} (trans)	$A_u + E_u$	$A_u + E_u$	$A_u + E_u$

[1] Total is the sum of the columns labelled "factor group".

[2] Sym. = symmetry, Rep. = irreducible representation of the point group, No. = number.

[3] Symmetry of the site occupied by the molecule in the crystal.

Table S2. Correlation table for β-WCl_6 (space group $P\bar{3}m1$ (D_{3d}^3) no. 164).

**Free molecule**		**Crystal (Z = 3)**				**Total** [1]
		**Site** [3] **(D_{3d})**	**Factor group (D_{3d}) 1 mol**	**Site** [3] **(C_{3v})**	**Factor group (D_{3d}) 2 mol**	
**Sym.** [2]	**Rep.**	**Rep.**	**Rep.**			
O_h	A_{1g} (ν1)	A_{1g}	A_{1g}	A_1	(A_{1g} + A_{2u})	2A_{1g} + A_{2u}
O_h	E_g (ν2)	E_g	E_g	E	(E_g + E_u)	2E_g + E_u
O_h	T_{1u} (ν3)	A_{2u} + E_u	A_{2u} + E_u	A_1 + E	(A_{1g} + A_{2u}) + (E_g + E_u)	A_{1g} + 2A_{2u} + E_g + 2E_u
O_h	T_{1u} (ν4)	A_{2u} + E_u	A_{2u} + E_u	A_1 + E	(A_{1g} + A_{2u}) + (E_g + E_u)	A_{1g} + 2A_{2u} + E_g + 2E_u
O_h	T_{2g} (ν5)	A_{1g} + E_g	A_{1g} + E_g	A_1 + E	(A_{1g} + A_{2u}) + (E_g + E_u)	2A_{1g} + A_{2u} + 2E_g + E_u
O_h	T_{2u} (ν6)	A_{1u} + E_u	A_{1u} + E_u	A_2 + E	(A_{2g} + A_{1u}) + (E_g + E_u)	A_{2g} + 2A_{1u} + E_g + 2E_u
O_h	T_{1g} (lib)	A_{2g} + E_g	A_{2g} + E_g	A_2 + E	(A_{2g} + A_{1u}) + (E_g + E_u)	2A_{2g} + A_{1u} + 2E_g + E_u
O_h	T_{1u} (trans)	A_{2u} + E_u	A_{2u} + E_u	A_1 + E	(A_{1g} + A_{2u}) + (E_g + E_u)	A_{1g} + 2A_{2u} + E_g + 2E_u

[1] Total is the sum of the columns labelled "factor group".

[2] Sym. = symmetry, Rep. = irreducible representation of the point group.

[3] Symmetry of the site occupied by the molecule in the crystal.

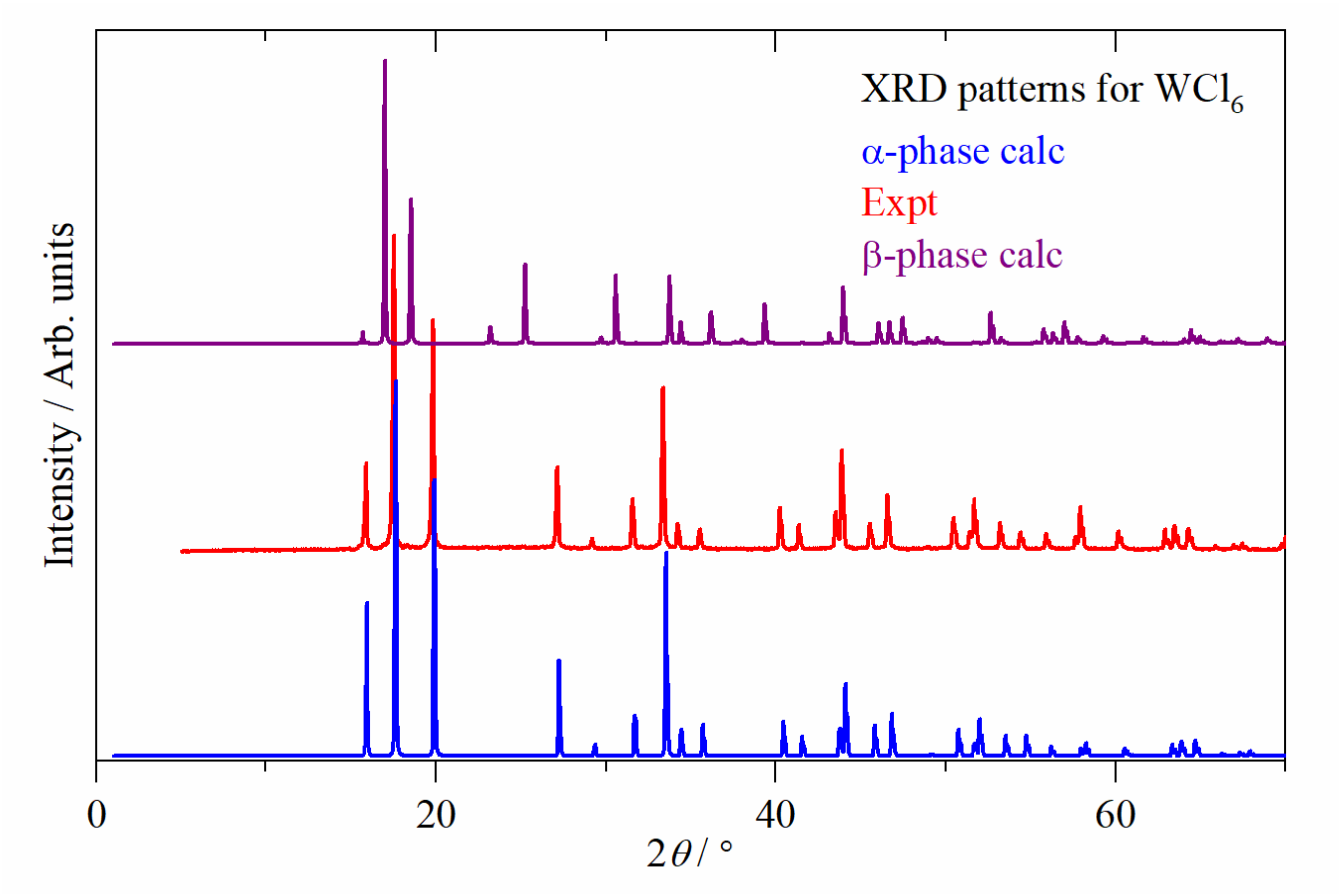

Figure S1. Comparison of powder XRD patterns for the WCl_6 sample used in this work (middle, red) with those calculated from the literature for α-WCl_6 (bottom, blue) and β-WCl_6 (top, purple). It is apparent that the sample is α-WCl_6.

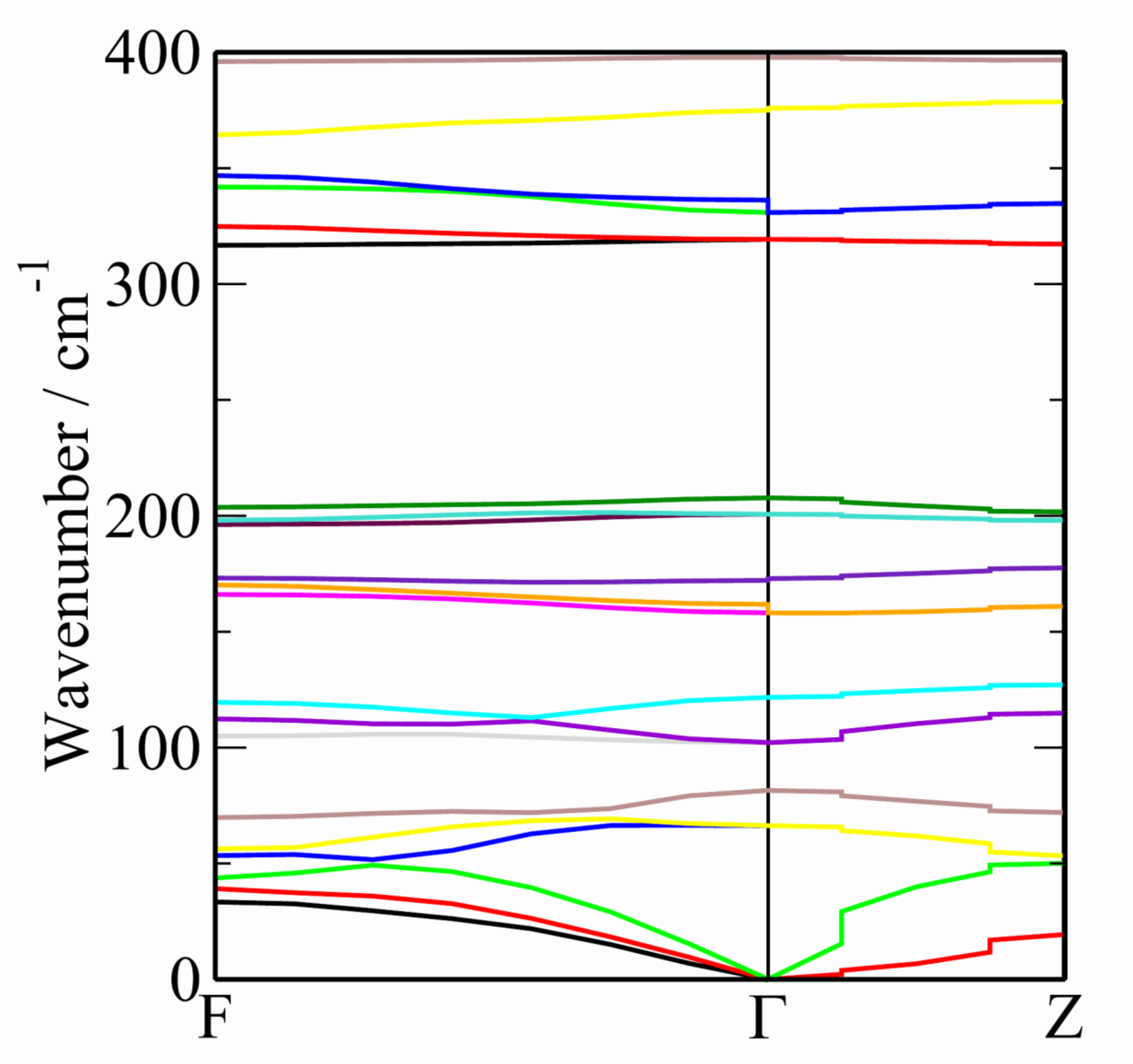

Figure S2. Phonon dispersion curves for α-WCl_6.

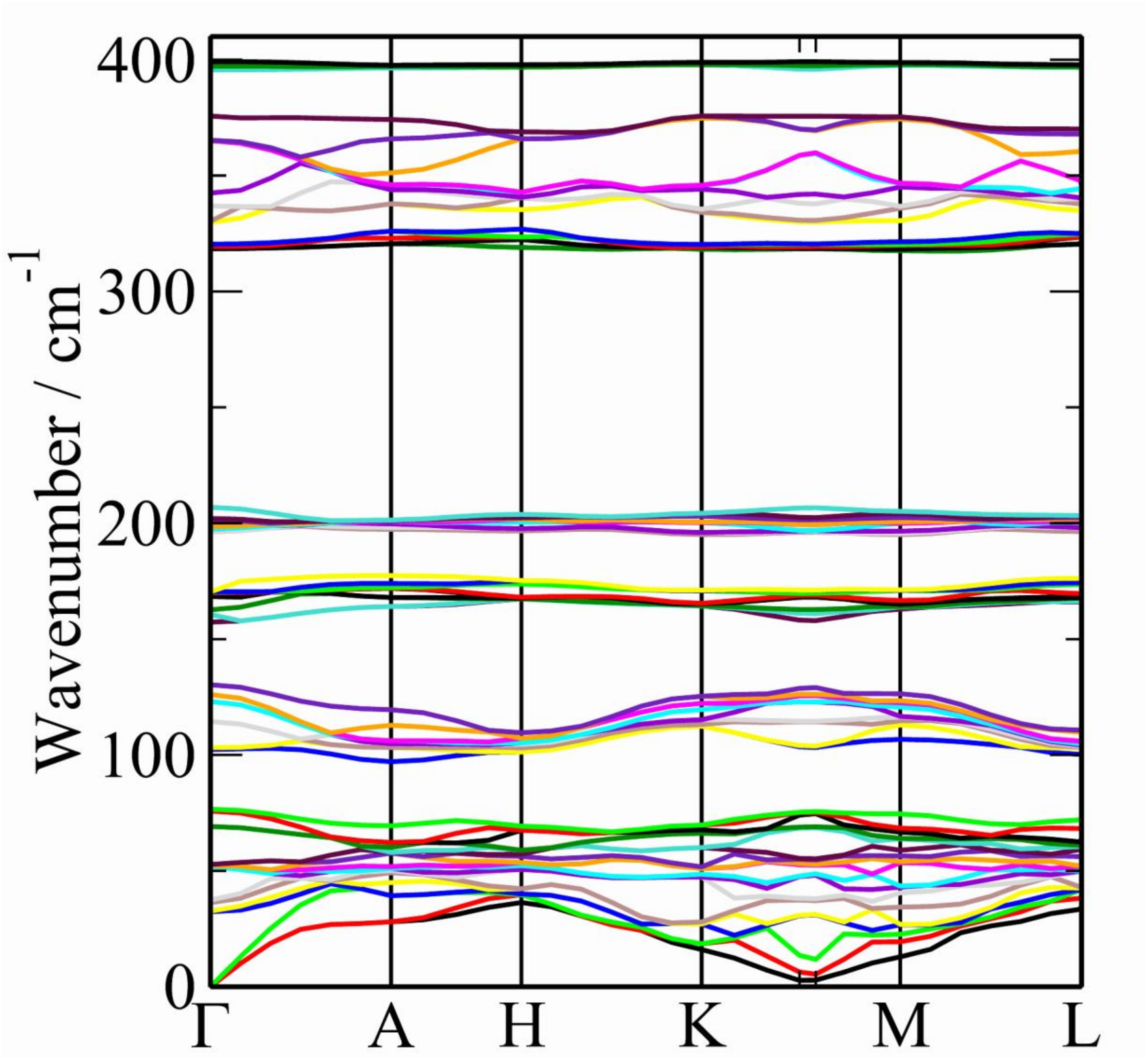

Figure S3. Phonon dispersion curves for β-WCl_6.

Table S3. Correlation table for WOCl4 (space group I4, no. 79)

Free molecule		Crystal (Z = 1)		Total [1]
		Site [3] (C_4)	Factor group (C_4)	
**Sym.** [2]	**Rep.**	**Rep.**		
C_{4v}	$A_1 + E$ (trans)	$A + E$	$A + E$	$A + E$
C_{4v}	$A_2 + E$ (lib)	$A + E$	$A + E$	$A + E$
C_{4v}	A_1 (ν_1)	A	A	A
C_{4v}	A_1 (ν_2)	A	A	A
C_{4v}	A_1 (ν_3)	A	A	A
C_{4v}	B_1 (ν_4)	B	B	B
C_{4v}	B_1 (ν_5)	B	B	B
C_{4v}	B_2 (ν_6)	B	B	B
C_{4v}	E (ν_7)	E	E	E
C_{4v}	E (ν_8)	E	E	E
C_{4v}	E (ν_9)	E	E	E

[1] Total is the sum of the columns labelled "factor group".

[2] Sym. = symmetry, Rep. = irreducible representation of the point group, No. = number.

[3] Symmetry of the site occupied by the ion in the crystal.

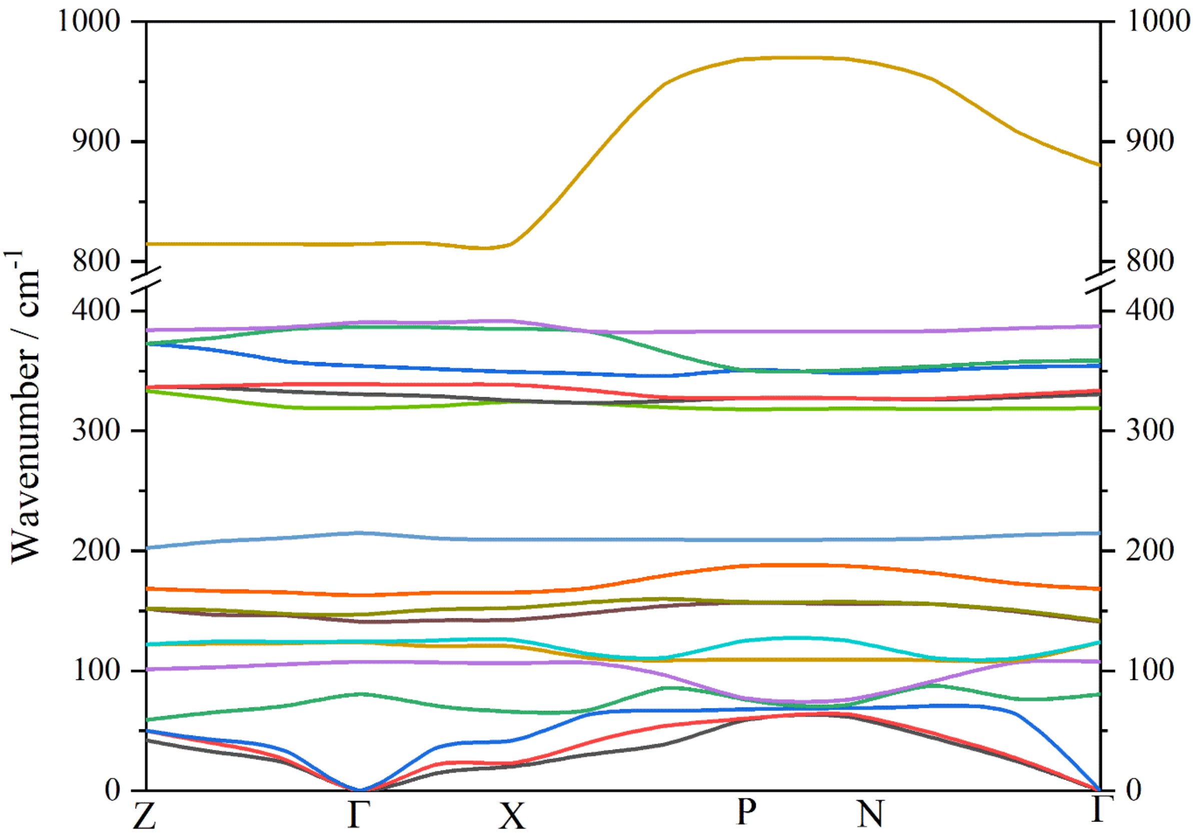

Figure S4. Phonon dispersion curves for $WOCl_4$.
